# Designing circulating tumor DNA-based interventional clinical trials in oncology

**DOI:** 10.1186/s13073-019-0634-x

**Published:** 2019-04-19

**Authors:** Daniel V. Araujo, Scott V. Bratman, Lillian L. Siu

**Affiliations:** 1 Division of Medical Oncology and Hematology, Princess Margaret Cancer Centre, University of Toronto, Toronto, Ontario Canada; 2 Department of Radiation Oncology, Princess Margaret Cancer Centre, University of Toronto, Toronto, Ontario Canada

## Abstract

Circulating tumor (ct) DNA is a powerful tool that can be used to track cancer beyond a single snapshot in space and time. It has potential applications in detecting minimal residual disease and predicting relapse, in selecting patients for tailored treatments, and in revealing mechanisms of response or resistance. Here, we discuss the incorporation of ctDNA into clinical trials.

## Circulating tumor DNA as a tool for tracking cancer

Advances in liquid biopsy technologies, such as the use of circulating tumor DNA (ctDNA), have empowered researchers to track cancer longitudinally through real-time monitoring. Increasingly, ctDNA is being integrated into clinical trials in order to evaluate its utility in detecting cancers before they are radiologically visible, in monitoring minimal residual disease (MRD) to predict relapse, in selecting patients for specific therapies, and in revealing mechanisms of treatment response or resistance. The validity of ctDNA as a predictive biomarker depends not only on the technical characteristics of the assay, but also on the coupling of ctDNA dynamics to clinical outcome so that they serve as a relevant biological surrogate. ctDNA exists as short fragments (150–200 base pairs) that are amenable to PCR- and next generation sequencing (NGS)-based analyses, with NGS offering greater multiplexing capabilities for mutation profiling. Beyond mutations, tools are now available to measure epigenetic features within ctDNA, including methylation; these tools may prove useful for cancer types that are associated with few recurrent mutations and for early detection and classification [1].

**Fig. 1:**
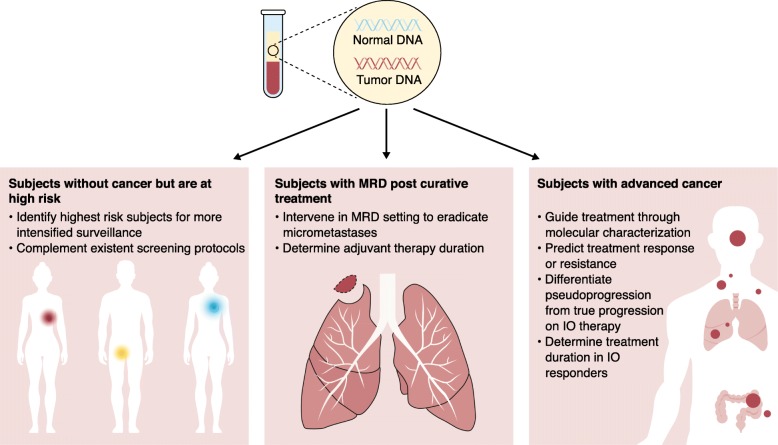
The application of ctDNA in clinical trials across different disease settings in oncology. IO, Immuno-oncology therapy; MRD, Minimal residual disease

Many factors influence the abundance and detectability of ctDNA in cancer patients. At diagnosis, anywhere from > 90 to < 0.1% of plasma DNA is tumor-derived [2]. Tumor type and location influence ctDNA levels, as do prior treatments; other potential confounders such as demographic, comorbidity and environmental factors are less well characterized. Mutations of interest may be present in subclones within the ctDNA, creating additional challenges for detection. Furthermore, ctDNA has a short half-life (of around 1 h) and its kinetics can be complex. For instance, an initial rise in ctDNA levels followed by subsequent clearance can be an early indication of therapeutic efficacy. Clinical trial designs that utilize treatment-related ctDNA changes as a prognostic biomarker or as a surrogate endpoint need to take into consideration relevant confounders and the timing of blood collection in order to ensure accurate interpretation of results. Interventional ctDNA-based clinical trials using predictive marker validation frameworks in various oncological settings are actively emerging (Fig. 1).

## Designing clinical trials in subjects without a cancer diagnosis

The use of ctDNA as a cancer screening tool in the general population is limited largely by its low sensitivity and cost effectiveness; the number of participants needed to screen to detect a true positive case is large. Thus, strategies to enrich for patient populations that are at sufficiently high risk of malignancy are important in ctDNA-based screening initiatives. Financial implications should also be considered in order to justify the implementation of a ctDNA screening strategy if clinical utility is demonstrated. An example of a risk-based ctDNA screening study is the prospective testing of circulating plasma Epstein-Barr virus (EBV) DNA in over 20,000 Chinese men from Hong Kong (aged between 40 to 62 years) to detect asymptomatic nasopharyngeal carcinoma (NPC) [3]. In this study, individuals with two consecutive positive ctDNA results were referred on to endoscopic examination and magnetic resonance imaging, which demonstrated the utility of using these samples for early detection. Another example of ctDNA testing in high-risk individuals is currently ongoing under the auspices of the Liquid Biopsy Program at the Princess Margaret Cancer Centre (trial number NCT03702309). This project enrolls healthy carriers (‘previvors’) of a germline pathogenic variant in hereditary cancer predisposition genes, such as *BRCA1/2*, *NF1* or *TP53*, and mismatch repair genes. Owing to their heightened cancer risk, these carriers may undergo risk-reduction surgeries such as mastectomy and/or intensive surveillance of some, but not all, organs. Given that the ‘first-hit’ (the germline mutation) is known and their tumorigenesis follows a predictable pattern, ctDNA analysis is an attractive complementary modality to current screening protocols in this population.

## Designing clinical trials in subjects after curative treatment

One of the most appealing clinical applications of ctDNA is to detect cancer recurrence in the MRD setting after definitive local or locoregional therapy, as it offers the opportunity to eradicate micrometastatic disease and maximize cure. Observational studies correlating the presence of ctDNA or specific genomic aberrations with disease outcome have shown a prognostic role across multiple tumor types, with positive ctDNA status typically preceding the occurrence of clinical relapse by a few months [4]. Clinical trials investigating therapeutic actions that are triggered by the detection of ctDNA in the MRD setting are being developed using frameworks for the validation of predictive markers.

### Interventional ctDNA-based studies in the MRD setting

In the marker-by-treatment interaction design framework, all patients are tested for the marker: marker-positive patients are randomized to investigational therapy versus control; marker-negative patients can undergo the same randomization as part of the study, or more often, are offered off-trial standard treatment. An example is a recently reported study in locoregionally advanced NPC that used testing of plasma EBV DNA after radiotherapy or chemoradiotherapy to select patients who have positive EBV DNA levels for randomization to adjuvant chemotherapy or observation [5]. In the marker-based strategy design framework, patients are randomized either to have marker testing and subsequent treatment assignment on the basis of the results or not to have marker testing (or blinded to testing results) and are offered standard care; an example is the ongoing DYNAMIC study in stage II colon cancer (trial number ACTRN12615000381583).

### ctDNA as a determinant for duration of adjuvant therapy

The optimal duration of adjuvant therapy, especially when using molecularly targeted therapy or immuno-oncology therapy (IO) after definitive surgery and/or radiotherapy, is often determined empirically rather than on the basis of biological rationale. In the case of IO, activation of memory T cells can promote sustained responses irrespective of treatment duration. Randomized studies that compare different adjuvant therapy durations generally have large sample sizes in order to demonstrate non-inferiority, and are often conducted by cooperative groups rather than by pharmaceutical sponsors. Clinical trials using ctDNA clearance to inform adjuvant treatment duration, in comparison to existent standard duration, would be of interest as they may help to reduce patient exposure to unnecessary toxicity and treatment-related cost.

## Designing clinical trials in patients with advanced cancer

The measurement of ctDNA in advanced cancer enables non-invasive access to genomic changes in the tumor that may guide therapeutic decisions. In some cases, predictive information can be obtained to select the optimal therapy, whereas in other cases, prognostic and pharmacodynamic information can provide a ‘headstart’ that allows intervention before clinical or radiological changes become manifest.

### Baseline ctDNA panels guiding treatment decisions

ctDNA data generated using high-throughput NGS panels can provide value by directly identifying known or new actionable mutations for genotype–drug matching. For example, ctDNA has been incorporated into standard of care as a less invasive alternative to tissue biopsy for detecting the *T790 M* mutation in *EGFR* mutant non-small cell lung cancer (NSCLC) patients who are progressing on first-generation tyrosine kinase inhibitors. If the panel size is sufficiently large, NGS data can also be used to calculate blood-based tumor mutational burden (bTMB) as a potential predictor of IO response, as demonstrated by retrospective analyses in NSCLC [6]. Clinical trials exploring the versatility of ctDNA-based high-throughput NGS genotyping, such as the ongoing B-FAST trial in NSCLC (NCT03178552) exemplifies these concepts, and patients are enrolled to four different molecularly defined cohorts on the basis of their ctDNA result.

### Early changes in ctDNA as a surrogate for treatment response

Early changes in ctDNA dynamics upon treatment can provide information about therapeutic efficacy, as demonstrated in a retrospective analysis of samples from the phase III PALOMA-3 trial in advanced estrogen-receptor-positive breast cancer. A decline in *PIK3CA* ctDNA levels compared to baseline after 15 days of treatment with palbociclib and fulvestrant was predictive of progression-free survival [7]. Several groups have demonstrated similar results in different tumor types, using diverse treatments. Notwithstanding the urgent need to standardize ctDNA methods and readouts for clinical translation, interventional trials using early ctDNA dynamics to predict treatment response and to avoid overexposure to ineffective drugs are in development.

### ctDNA to differentiate true progression from pseudo-progression

For patients on IO therapy, validated methods are lacking to distinguish patients with disease progression from those experiencing pseudo-progression resulting from immune cell infiltration in the tumor microenvironment. In a cohort of 125 melanoma patients treated with PD-1 blockade, retrospective ctDNA analysis of *BRAF*/*NRAS* mutations by Droplet Digital PCR (dd-PCR) successfully identified all 9 patients who had pseudo-progression [8]. Prospective ctDNA assessments may help to address the challenges in these clinical decisions and to direct patients with true progression to alternative therapeutic options.

### ctDNA as a determinant of duration of IO treatment

Akin to the adjuvant setting, the optimal duration of therapy, especially IO, in those who have objective tumor response or prolonged disease stabilization is unclear and may be disease specific. Fewer than 10% of metastatic melanoma patients completing two years of PD-1 blockade relapse afterwards [9]. Conversely, one-year duration of PD-1 blockade in advanced NSCLC had results that were inferior to those of continuous treatment [10]. In line with their role in predicting treatment response, changes in ctDNA dynamics may provide insights into this clinical issue.

## Conclusions

In the era of precision medicine, ctDNA-based interventional studies represent a new frontier, targeting molecular changes in cancer beyond a single snapshot in space and time. Innovative strategies are being sought to incorporate ctDNA or other liquid biopsies into clinical trials in order to validate their role as a predictive biomarker across different tumor types and various disease settings. These studies can contribute important information to multi-omic and longitudinal evaluations that can best inform dynamic changes in cancer.

## Abbreviations

ctDNA: Circulating tumor DNA
EBV: Epstein-Barr virus
IO: Immuno-oncology therapy
MRD: Minimal residual disease
NGS: Next generation sequencing
NPC: Nasopharyngeal carcinoma
NSCLC: Non-small cell lung cancer
